# Efficacy of praziquantel and artemisinin derivatives for the treatment and prevention of human schistosomiasis: a systematic review and meta-analysis

**DOI:** 10.1186/1756-3305-4-201

**Published:** 2011-10-17

**Authors:** Rong Liu, Hui-Fen Dong, Yi Guo, Qin-Ping Zhao, Ming-Sen Jiang

**Affiliations:** 1 Department of Medical Parasitology, School of Basic Medical Science, Wuhan University, Wuhan 430071, People's Republic of China; 2 Department of Epidemiology, School of Public Health, Wuhan University, Wuhan 430071, People's Republic of China

**Keywords:** human schistosomiasis, praziquantel, artemether, artesunate, efficacy, meta-analysis

## Abstract

**Background:**

Praziquantel has been used as first-line drug for chemotherapy of schistosomiasis since 1984. Besides praziquantel, artemether and artesunate have also been used for the control of this infectious disease since late 1990s. In this article, we conducted a systematic review and meta-analysis to evaluate the antischistosomal efficacy of different medication strategies including monotherapy or combination therapies of these drugs.

**Results:**

A number of 52 trials from 38 articles published in peer-reviewed journals before July 2011 were selected for analysis after searching the following literature databases: the Cochrane Library, PubMed/Medline, ISI Web of Science, Chinese Biomedicine Literature Database, and China National Knowledge Infrastructure. Our meta-analyses showed that a dosage of 30-60 mg/kg praziquantel compared with placebo produced a protection rate of about 76% (95% CI: 67%-83%) for treating human schistosomiasis, which varied from 70% to 76% with no significant differences among the subspecies *S. haematobium*, *S. japonicum *or *S. mansoni*. Protection rates were higher when praziquantel doses were elevated, as concluded from the nRCTs results: the protection rate of praziquantel at 40 mg/kg was 52% (95% CI: 49%-55%), and it increased to 91% (95% CI: 88%-92%) when the dosages were elevated to 60/80/100 mg/kg divided two or more doses. Multiple doses of artemether or artesunate over 1- or 2-week intervals resulted in protection rates of 65% to 97% for preventing schistosomiasis, and increased doses and shorter medication intervals improved their efficacies. Praziquantel and artemisinin derivatives (artemether or artesunate) in combination resulted in a higher protection rate of 84% (95% CI: 64%-91%) than praziquantel monotherapy for treatment. praziquantel and artesunate in combination had a great protection rate of 96% (95% CI: 78%-99%) for preventing schistosomes infection.

**Conclusions:**

According to the results, praziquantel remains effective in schistosomiasis treatment, and multiple doses would improve its efficacy; meanwhile, praziquantel is also a good drug for preventing acute schistosomiasis morbidity. It's better to use multiple doses of artemether or artesunate with 1- or 2-week intervals for prevention against schistosome infection. Praziquantel and artemether or artesunate in combination perform better in treatment than praziquantel monotherapy, and they are especially suitable for treating the patients with repeated exposure to infected water.

## Background

Schistosomiasis, an infectious disease caused by parasitic trematodes (schistosomes) dwelling in the host's mesenteric portal system, is a great public health problem in tropical and subtropical regions. The disease causes health and labor loss, and finally a significant reduction in socioeconomic benefits. Approximately 207 million people (more than 97% in Africa) are infected, and 779 million (85% in Africa) are at risk of being infected in 76 endemic countries worldwide, leading to the loss of about 4.5 million disability-adjusted life years (DALYs) [1-4]. Thus schistosomiasis control remains a challenge in endemic regions [4-7].

There are five schistosome species parasitizing in humans: *Schistosoma japonicum*, *S. mansoni*, *S. haematobium*, *S. mekongi*, and *S. intercalatum*. *S. japonicum *is transmitted by the amphibian snail *Oncomelania *and causes intestinal and hepatosplenic schistosomiasis in the People's Republic of China, Philippines, and Indonesia; *S. mansoni*, transmitted by *Biomphalaria *snails, causes intestinal and hepatic schistosomiasis in Africa, the Arabian peninsula, and South America; *S. haematobium*, transmitted by *Bulinus *snails, causes urinary schistosomiasis in Africa and the Arabian peninsula. *S. mekongi *and *S. intercalatum *are only of local importance [1-3,8,9].

In the mid-1980s, the WHO recommended schistosomiasis control strategies for humans by focusing on the large-scale population-based and repeated chemotherapy, which is still the key strategy today [10]. The chemotherapy for human schistosomiasis is to take antischistosomal drugs for prevention of morbidity in high-risk population (i.e. chemoprophylaxis by preventing the young schistosomes - schistosomula developing into adult egg-laying worms or killing them), and for treatment of patients by eliminating adult worms, whose eggs deposited in human tissue (e.g. liver and intestinal wall) and caused pathogenesis. For schistosomiasis treatment three drugs have been used, which differ in their effects on schistosome species: metrifonate (targeting *S. haematobium*), oxamniquine (targeting *S. mansoni*), and praziquantel (PZQ) (for all human species). Due to its broader spectrum, PZQ has finally become the first-line medicine. In recent years, however, the potential resistance problem of schistosomes to PZQ has come into concern, which may necessitate searching for alternatives [4,11-15]. Among those are artemether (AM) and artesunate (AS), artemisinin derivatives (ARTs) with anti-schistosomal potential which was first described in the People's Republic of China in the early 1980s. ARTs were approved as schistosomiasis prevention drugs by the Chinese Ministry of Health in 1996 [16]. They are active against *S. japonicum*, *S. mansoni *and *S. haematobium*, mainly targeting the immature, pre-adult stage, the schistosomulum [17-21].

Only few publications, however, can be found with comprehensive and statistical assessments of the efficacy of these drugs controlling human schistosomiasis. To get a more detailed overview on the efficacy of PZQ and ARTs for human schistosomiasis treatment and prevention, we collected a large group of studies published in peer-reviewed journals, and conducted meta-analyses in categories to assess their antischistosomal efficacy comprehensively, including single drug monotherapy and drugs in combination against *S. japonicum*, *S. mansoni*, *S. haematobium *and *S. mekongi*.

## Methods

### Search strategy and data sources

We conducted a computer-aided search of the literature about anti-schistosomal efficacies assessments of PZQ, AM and AS used alone or in combination for human schistosomiasis. Source databases were the Cochrane Library (Issue 7, 2011), PubMed/Medline (1966 to July 2011), ISI Web of Science (1975 to July 2011), Chinese Bio-Medicine Literature Database (CBM, 1979 to July 2011) and China National Knowledge Infrastructure (CNKI, 1994 to July 2011). A review of European and American "grey literature" databases (NTIS, SIGLE) were also conducted. The terms and medical subject headings (MeSH) used in retrieving citations were "schistosom*" (* means the inclusion of all words with the preceding radical), "praziquantel", "artemisinin", "artemether", "artesunate", "chemotherapy". The retrieval formula was: schistosom* AND (praziquantel OR artemether OR artesunate). The searches were performed mainly in Chinese and English with a limitation to human participants. One reviewer (LR) identified relevant studies by screening titles and abstracts; a manual search was performed systematically using the authors' reference lists. The full-texts of potentially relevant studies were further evaluated by three reviewers (RL, HFD and YG).

### Criteria of inclusion and exclusion

The inclusion criteria were: (i) independent studies assessing the antischistosomal efficacy of PZQ, AM and AS, administrated alone or in combination for human schistosomiasis treatment and prevention; (ii) the year of the study conducted or published was reported; (iii) the sample size was reported; (iv) the same drugs' efficacy evaluation indicators between experimental populations and control populations, i.e. reporting parasitological outcome eggs-positive or eggs-negative by Kato-Katz thick stool smears technique and/or miracidia hatching tests for detecting eggs of *S. japonicum*, *S. mansoni*, and *S. mekongi *or urine filtration for detecting eggs of *S.haematobium *after approximately 3-4 weeks post-treatment, which was recommended as the standard method for schistosomiasis parasitological diagnosis by WHO in 1980 [10]; or reporting emergence or absence of acute schistosomiasis in the trials of assessing some drug's efficacy in controlling morbidity; (v) the studies were either randomized controlled trials (RCTs) or non-randomized control trials (nRCTs); (vi) with raw data which could be changed into relative risk (RR) and 95% CI RR and 95% CI were reported.

**Figure 1:**
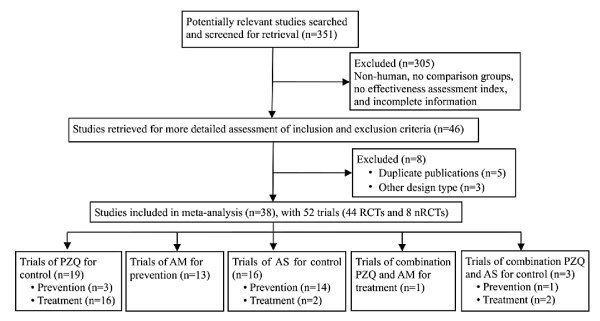
**Flow diagram showing the articles selection process for present meta-analyses of the efficacy of PZQ and ART (AM and AS) administrated alone or in combination for human schistosomiasis treatment or prevention**. Individual searches don't add up to 351 because some articles could be found simultaneously in multiple literature databases.

Exclusion criteria were: (i) study participants were not human; (ii) without control group; (iii) incomplete information; (iv) duplicate publications; (v) studies described only results without detailed background and method introduction; (vi) reviews. Figure 1 summarizes the studies selection process.

### Data extraction and methodological/quality assessment

The following information was independently extracted by two reviewers (RL and YG) and was checked together, discrepancies were solved through discussion. The extracted information included: first author's name and year of publication, test sites (i.e. where trials were implemented), time (i.e. when trials were implemented), participants, *schistosoma *species, interventions, diagnostics methods, follow-up time, raw dichotomous data of efficacy assessment (NO. of positive/NO. of diagnosed), RRs and their 95% CIs, type of study design (RCT or nRCT), and intervention purposes (prevention or treatment).

The quality of included RCTs was assessed by examining whether there is randomization, blinding, and information about follow-up and dropouts/withdrawals of participants according to the guidance of the methodological quality assessment of RCTs in the *Cochrane Handbook for Systematic Reviews of Interventions 4.2.6 *and the Jadad scoring criteria [22,23]. The score for quality scale ranges from 0 to 5 points, the higher the score, the higher the quality of trial; and a trial with a Jadad score ≥3 has been considered to be of ample quality.

### Diseases, interventions and outcomes

Schistosomiasis japonica, schistosomiasis mansoni, schistosomiasis haematobia, and schistosomiasis mekongi were included in this meta-analysis. The participants included schoolchildren, villagers, schistosomiasis patients, fishermen, and people or soldiers participating in fighting against floods.

The participants of experimental groups took antischistosomal drugs such as PZQ, AM, or AS alone or in combination for treatment (to remove the adult worms from schistosomiasis patients by PZQ) or prevention (to control morbidity in high-risk population by ART or PZQ). Control groups took the same dose of placebo or PZQ (of the combination therapy trials) or nothing. The chemotherapeutic outcome evaluation was parasitological cure, which was defined as eggs-positive or eggs-negative, or emergence or absence of acute schistosomiasis symptoms.

### Data synthesis and statistical analysis

Meta-analyses were conducted in categories of PZQ, AM, or AS alone and in combination, respectively. RRs based on dichotomous data were set as statistical indicators. Subgroup analyses were conducted according to different design types, *schistosoma *species, time of medication and dosages. The protection rates were calculated based on the summary RRs. All the statistical analysis work was performed using the statistical package ***Review Manager 4.3 ***software (Cochrane Collaboration, Oxford, UK) and ***Stata/SE 11 ***(*Stata *^® ^Corporation, Texas, USA) for Egger's publication bias test by LR. The fixed-effects model was used to combine study-specific RRs, when there was no significant heterogeneity among studies. Otherwise, the random-effects model was used.

### Publication bias and sensitivity analysis

In order to examine the reliability and stability of our meta-analyses, publication bias was assessed by means of both funnel plot (qualitative) and Egger's publication bias test (quantitative). In addition, sensitivity analysis was performed by excluding some trials with different schistosome species (as the parasitological diagnosis method used for *S. haematobium *is different from that of other species) or with larger sample size.

## Results

### Studies selected

Overall, 38 articles with 52 trials met the inclusion criteria and were finally used for this meta-analysis. Figure 1 shows the studies' selection process: 19 trials on PZQ's efficacy evaluation (9 RCTs and 7 nRCTs on treatment, 2 RCTs and 1 nRCT on prevention) [24-37]; 12 RCTs on AM's efficacy for prevention [38-49]; 16 RCTs on AS's efficacy (14 RCTs for prevention and 2 RCTs for treatment) [24,30,50-59], 6 RCTs out of Zhang et al.'s study [55] of AS's efficacy in preventing *S. japonicum *infections reported the duplications of studies from Liu et al. [58], Yi et al. [56], Xu et al. [50,51], and Liu et al. [57] and were excluded; one RCT on PZQ and AM in combination for treatment [60]; 3 RCTs on PZQ and AS in combination (2 RCTs for treatment and one for prevention) [24,30,61]. A number of 5 studies contained 2 or more independent trials [34,35,37,38,55]. Further 5 studies [62-66] were excluded for duplicate publication, and 3 studies for different design types [67-69].

### Study characteristics and methodological quality

Studies were conducted in areas that are endemic for human schistosomiasis, including Nigeria, Burkina Faso, Niger, KwaZulu/Natal of South Africa, Côte d'Ivoire, Gabon, Philippines, Tanzania, Brazil, and China (see Additional file 1, Table S1). PZQ dosages applied ranged from a single oral dose of 30-60 mg/kg or divided (2-3) dosages in RCTs-designed studies, and only one trial reported about participants who had received a single oral dose of PZQ at 20 mg/kg [35]. For nRCTs about PZQ's efficacy, there were several types of drug administration i.e. a single oral dose of 40 mg/kg or 60 mg/kg, multiple (2-3) oral doses of the same concentrations, two doses of 40 mg/kg, and two doses of 30 mg/kg added by another dose of 40 mg/kg. The dosage of AM given was 6 mg/kg/day, ranging from 2 doses to 13 doses over about 15-day intervals, 3-week intervals, or one-month interval, for preventing schistosomiasis morbidity. AS was applied by two medication types, 4 mg/kg/day over 3 consecutive days for treatment, or 3-14 doses of 6 mg/kg/day over 1- or 2-weeks intervals for prevention. For PZQ and AM in combination participants of the experimental group received a single oral dose of 60 mg/kg PZQ and 6 mg/kg/day AM [60]; for PZQ and AS in combination for treating schistosomiasis haematobia, participants received a single oral dose of 40 mg/kg PZQ and a dose of 4 mg/kg/day AS [24,29]. Participants in the control groups received just a single oral dose of 40 or 60 mg/kg PZQ and AM placebo or AS placebo. In another study with PZQ and AS in combination to treat schistosomiasis japonica by soldiers participating in fighting against floods, participants of the experimental group received 1200 mg PZQ once divided into two doses and AS 300 mg once for every 7 days, participants in the control group received nothing [61]. The follow-up time post-treatment differed from studies ranging from 2 weeks to more than 2 years, most of which ranged from 4 weeks to half a year. Table 1 summarizes the Jadad scores of the included RCTs. Among the 29 RCT-designed studies, only 5 studies [29,40,41,43,51] have described the randomization method; one study [37] did not include blinded allocation or outcome measurements, and 11 studies [24,38,43,49,51-55,57,58] had no description of withdrawals or dropouts. Thus 28 of the included RCTs were rated as providing good methodological quality based on a Jadad score of 3-5, and only one study [37] had a Jadad score of 2. The nRCTs without quality assessment were analyzed separately from the RCTs.

**Table 1:** Assessment of methodological quality of the included RCTs by Jadad scoring criteria*

Trial	Randomized†	Double-blinded§	A description of withdrawals or dropouts	Jadad score
Inyang-Etoh PC, 2009	1	2	0	3
Borrmann S, 2001	2	2	1	5
Santos AT, 1979	1	2	1	4
McMahon JE, 1979	1	1	1	3
Katz N, 1979	1	2	1	4
Liu ZG, 1997	1	0	1	2
Song Y, 2006	1	2	0	3
Li YS, 2005	1	2	1	4
N'Goran EK, 2003	2	2	1	5
Utzinger J, 2000	2	2	1	5
Tian ZY, 1999	2	2	0	4
Huang AS, 1999	1	2	1	4
Song Y, 1998	1	2	1	4
Xu MS, 1997	1	2	1	4
Tian ZY, 1997	1	2	1	4
Wang JL, 1997	1	2	1	4
Xiao SH, 1996	1	2	0	3
Xiao SH, 1995	1	2	1	4
Xu MS, 2001	1	2	0	3
Cui JF, 2001	1	2	0	3
Sun MX, 2000	1	2	0	3
Zhang SJ, 2000	1	2	0	3
Yi ZH, 2000	1	2	1	4
Liu HY, 1999	1	2	0	3
Xu MS, 1998	2	2	0	4
Liu ZD, 1996	1	2	0	3
Xu MS, 1996	1	2	1	4
Wu LJ, 1995	1	2	1	4
Hou XY, 2008	1	2	1	4

* Range 0~5 (the higher the Jadad score is, the higher the quality of study is).

† Represents generation of allocation sequence.

§ Represents allocation concealment.

### Meta-analysis

**Figure 2:**
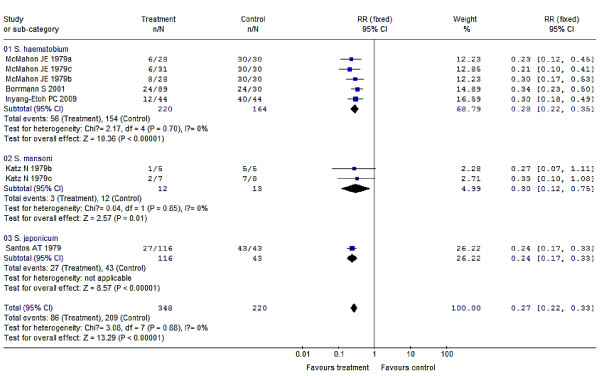
**Forest plots showing the efficacy of PZQ (30-60 mg/kg) for schistosomiasis treatment (RCTs)**. n/N = number examined as positive outcome (or not cured) over number of participants who were examined. Sub-groups with trials with patients infected by different *Schistosoma *species (01: *S. haematobium*, 02: *S. mansoni*, and 03: *S. japonicum*) were separately combined. The *P *value of each test for heterogeneity was ≥0.05, thus the fixed-effect model was used to combine trial-specific RRs of each sub-group and the total pooled RR, and its 95% CI were calculated by combing all sub-groups. No statistically significant difference among pooled RRs of sub-groups about different species was observed.

**Figure 3:**
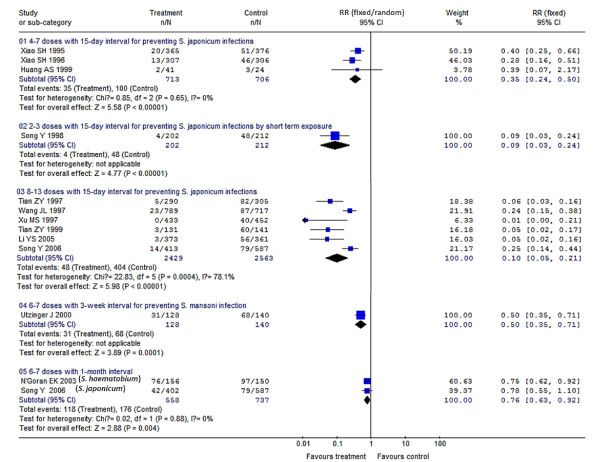
**Forest plots showing the efficacy of AM using 6 mg/kg for preventing schistosome infection (RCTs)**. The trials were stratified into sub-groups based on dosages and *Schistosoma *species. (01) 4-7 doses by 15-day intervals for preventing *S. japonicum *infection (pooled RR was synthesized by the fixed-effect model); (02) 2-3 doses by 15-day intervals for preventing *S. japonicum *infection of short term exposure; (03) 8-13 doses by 15-day intervals for preventing *S. japonicum *infection (pooled RR was synthesized by random-effect model); (04) 6-7 doses by 3-week intervals for preventing *S. mansoni *infection; (05) 6-7 doses by 1-month intervals for preventing schistosome infection - *S. haematobium *and *S. japonicum *(pooled RR was synthesized by the fixed-effect model).

**Figure 4:**
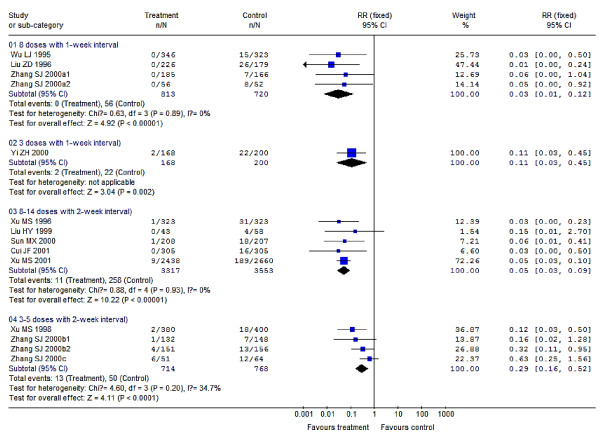
**Forest plots showing the efficacy of AS using 6 mg/kg for preventing *S. japonicum *infection (RCTs)**. The trials were stratified into sub-groups based on different dosages and time intervals between every two adjacent doses. (01) 8 doses by 1-week intervals; (02) 3 doses by 1-week intervals; (03) 8-14 doses by 2-week intervals; (04) 3-5 doses by 2-week intervals. The *P *values of test for heterogeneity of subgroups 01, 03 and 04 were >0.05 each, thus the pooled RRs were synthesized by the fixed-effect model.

**Figure 5:**
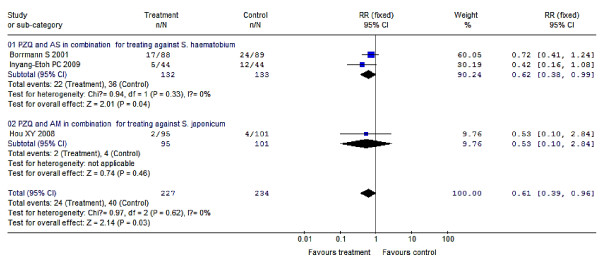
**Forest plots showing the efficacy of PZQ and ARTs (AM or AS) in combination for schistosomiasis treatment**. (01) PZQ and AS in combination for treating against *S. haematobium *or *S. japonicum*; (02) PZQ and AM in combination for treating against *S. japonicum*. The pooled RR of 0.61 (95% CI: 0.39-0.96) was determined by combing the two subgroups as the *P *value of overall heterogeneity test was 0.62.

Compared to results using a placebo, a single dose of 30-60 mg/kg PZQ provided a protection rate of 76% (95% CI: 67%-83%) for treatment of schistosomiasis japonica, which was higher as point estimation than its protection rate against schistosomiasis haematobia [72% (95% CI: 65%-78%)] or schistosomiasis mansoni [70% (95% CI: 25%-88%)]. But, the differences of protection rates among the three species are of no statistical significance with regard to their 95% CIs, and their overall pooled protection rate is 73% (95% CI: 67%-78%); meanwhile, the increasing dosage within 30-60 mg/kg gives no significant improvement in efficacy. Compared to this, 20 mg/kg PZQ seems to be less effective resulting in a protection rate of just 16% (95% CI: -81%-61%). For PZQ's efficacy during nRCTs, dosages of 60-100 mg/kg (i.e. 60/(40 × 2 doses)/(30 × 2 doses + 40) mg/kg) provide better efficacies than a single dose at 40 mg/kg, their protection rates were 91% (95% CI: 88%-92%) vs. 52% (95% CI: 49%-55%), respectively. In addition, several doses of PZQ at 40 mg/kg of both two RCTs and one nRCT produced great effects on preventing schistosomiasis morbidity, their protection rates were 98% (95% CI: 93%-99%) and 100%, respectively. Multiple (6-7) doses of 6 mg/kg AM applied in 1-month intervals resulted in protection rates of 24% (95% CI: 8%-37%) with respect to preventing *S. japonicum *infections, which increased to 50% (95% CI: 29%-65%) when the time interval was shortened to 3-week intervals; 4-7 doses of 6 mg/kg AM applied in 15-day intervals led to protection rates of 65% (95% CI: 50%-76%) in preventing *S. japonicum *infections, which increased significantly to 90% (95% CI: 79%-95%) when the dosages were increased to 8-13 doses. In addition, 2-3 doses of 6 mg/kg AM with 15-day intervals produced great effects on preventing against *S. japonicum *infection in persons of short term exposure, who e.g. participated in fighting against floods, here, the protection rate was 91% (95% CI: 76%-97%). 3 doses of 6 mg/kg AS given during 1-week intervals used to prevent *S. japonicum *infection resulted in protection rates of 89% (95% CI: 55%-97%), which rose to 97% (95% CI: 88%-99%) when the dosages increased to 8 doses; 3-5 doses of 6 mg/kg AS applied in 2-week intervals used to prevent *S. japonicum *infection led to a protection rate of 71% (95% CI: 48%-84%), which rose significantly to 95% (95% CI: 91%-97%) when the dosages increased to 8-14 applications. Two studies showed no significant effects of AS treatments compared to a placebo, with a protection rate of 45% (95% CI: -78%-83%) which spanned across zero. PZQ and ART (AM or AS) in combination used for schistosomiasis treatment resulted in a better efficacy than PZQ treatment alone, with a pooled RR of 0.61 (95% CI: 0.39-0.96) and a protection rate of 39% (95% CI: 4%-61%), which was converted to 84% (95% CI: 64%-91%) after the control PZQ was transformed into placebo based on the RCTs' result of PZQ at 30-60 mg/kg for treatment. PZQ and AS in combination had a great effect on prevention against *S. japonicum *infection with a protection rate of 96% (95% CI: 78%-99%). All of these results are summarized in detail in Figure 2, 3, 4 and 5 and Table 2.

**Table 2:** Estimates of summary RRs and protection rates of the antischistosomal drugs' efficacy

Studies included	Trials (n)*	**Participants (N**_**1**_**/N**_**2**_**)**^**Δ**^	**Pooled RRs (95% CI)**^**▲**^	Protection rates % (95% CI)	Heterogeneity test, *P *values
**PZQ for treatment**	**16**	**4108/5331§**	-	-	-
• RCT designed (20 mg/kg) against *S. mansoni*	1	4/2	0.84 (0.39, 1.81)	16 (-81, 61)	-
• RCT designed (30-60 mg/kg)	8	348/220	0.27 (0.22, 0.33)	73 (67, 78)	0.88
•*S. haematobium*	5	220/164	0.28 (0.22, 0.35)	72 (65, 78)	0.70
•*S. mansoni*	2	12/13	0.30 (0.12, 0.75)	70 (25, 88)	0.85
•*S. japonicum*	1	116/43	0.24 (0.17, 0.33)	76 (67, 83)	-
• nRCT designed (40 mg/kg)	3	2375/2847	0.48 (0.45, 0.51)	52 (49, 55)	<0.01
• nRCT designed (60/(40 × 2)/(30 × 2 + 40) mg/kg)	4	1381/2262	0.09 (0.08, 0.12)	91 (88, 92)	0.17
**PZQ for prevention against *S. japonicum***	**3**	**2405/121**	-	-	-
• RCT designed	2	155/61§	0.02 (0.01, 0.07)	98 (93, 99)	0.15
• nRCT designed	1	2250/60	0.00 (0.00, 0.00)	100	-
**AM for prevention (6 mg/kg) (RCTs)**	**13**	**4030/3771§**	-	-	-
• 2-3 doses by 15-day interval against *S. japonicum *by short term exposure during fighting against floods	1	202/212	0.09 (0.03, 0.24)	91 (76, 97)	-
• 4-7 doses by 15-day interval against *S. japonicum*	3	713/706	0.35 (0.24, 0.50)	65 (50, 76)	0.65
• 8-13 doses by 15-day interval against *S. japonicum*	6	2429/2563	0.10 (0.05, 0.21)	90 (79, 95)	<0.01
• 6-7 doses by 3-week interval against *S. mansoni*	1	128/140	0.50 (0.35, 0.71)	50 (29, 65)	-
• 6-7 doses by 1-month interval against *S. haematobium *and *S. japonicum*	2	558/737	0.76 (0.63, 0.92)	24 (8, 37)	0.88
**AS (6 mg/kg) for preventing *S. japonicum *infection (RCTs)**	**14**	**5012/5241**	-	-	-
• 3 doses by 1-week interval	1	168/200	0.11 (0.03, 0.45)	89 (55, 97)	-
• 8 doses by 1-week interval	4	813/720	0.03 (0.01, 0.12)	97 (88, 99)	0.89
• 3-5 doses by 2-week interval	4	714/768	0.29 (0.16, 0.52)	71 (48, 84)	0.20
• 8-14 doses by 2-week interval	5	3317/3553	0.05 (0.03, 0.09)	95 (91, 97)	0.93
**AS (4 mg/kg × 3 doses) for treating S. haematobia (RCTs)**	**2**	**132/74**	**0.55 (0.17, 1.78)**	**45 (-78, 83)**	**<0.01**
**PZQ + AM/AS for treatment (RCTs)**	**4**	**227/234**	**0.61 (0.39, 0.96)**	**84 (64, 91) †**	**0.62**
• PZQ + AM against *S. japonicum*	1	95/101	0.53 (0.10, 2.84)	86 (6, 98) †	-
• PZQ + AS against *S. haematobium*	2	132/133	0.62 (0.38, 0.99)	83 (62, 92) †	0.33
PZQ + AS for prevention against *S. japonicum *(nRCT)	1	1362/112	0.04 (0.01, 0.22)	96 (78, 99)	-

* There are 39 studies with53 trials included as some of the studies have multiple drugs trials.

Δ N_1_/N_2_= the number of participants in test group/the number of participants in control group.

▲ Pooled RRs (95% CI) are presented which were calculated by fixed-effects model when *P *value ≥0.05 of heterogeneity test, and by random-effects model if not.

† Their average protection rates and 95% CI were calculated when the drugs given to the controls were transformed into placebo (based on the RCTs results of PZQ 30-60 mg/kg for treatment).

§ The sum of participants in subgroups doesn't equal the true total because some studies [35,39,64] have the same control participants.

### Publication bias and sensitivity analysis

**Figure 6:**
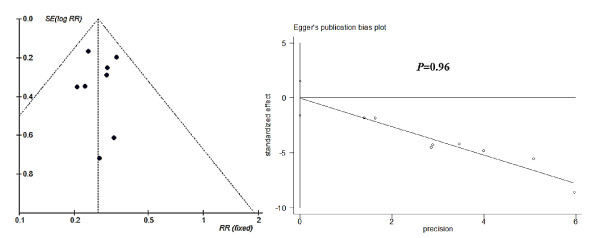
**Funnel plot and Egger's publication bias test for RCTs of PZQ's efficacy on schistosomiasis treatment**. The pooled log-RR for these trials is shown with a dashed vertical line, and the dashed slash lines distributed in both sides are the cutoff values of 95% CI of pooled log RR. The Egger's publication bias test showed that no publication bias exists (*P *= 0.96).

**Figure 7:**
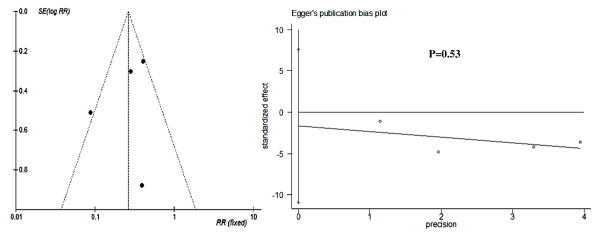
**Funnel plot and Egger's publication bias test for RCTs (2-7 doses by 15-day intervals) of AM's efficacy on preventing *S. japonicum *infection**. There is one point (representing one trial) outside the cutoff values of 95% CI of pooled log RR. The Egger's publication bias test showed that no publication bias exists (*P *= 0.53).

**Figure 8:**
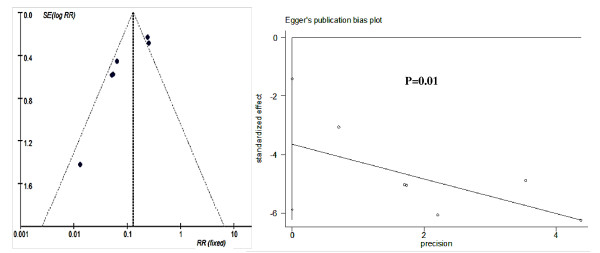
**Funnel plot and Egger's publication bias test for RCTs (8-13 doses in 15-day intervals) of AM's efficacy on preventing *S. japonicum *infection**. There are two points (represented two trials) distributing outside the cutoff values of 95% CI of pooled log RR. The Egger's publication bias test showed that a publication bias may exist (*P *= 0.01).

**Figure 9:**
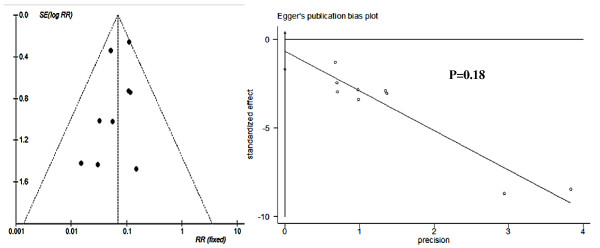
**Funnel plot and Egger's publication bias test for RCTs of AS's efficacy on preventing *S. japonicum *infection**. The Egger's publication bias test showed that no publication bias exists (*P *= 0.18).

Funnel plots and Egger's test results of publication bias estimation of each subgroup based on study-specific RRs showed that in most subgroups the included trials are almost evenly distributed around the vertical axis of pooled RR and within the 95% CI of pooled log RR (Figures 6,7, 8 and 9). This indicated that no evidence of a publication bias exists, except for the subgroup RCTs-designed of 8-13 doses of 6 mg/kg AM during 15 day-intervals used for prevention against *S. japonicum *infection. The *P *value of Egger's test was 0.01, which indicated that a publication bias may exist. In addition, sensitivity analyses indicated there were no significant changes in the results of RRs' estimation and their 95% CIs between the restricted data sets and their combined overall values in each subgroup (Table 3). Thus the results of our meta-analyses are stable and credible.

**Table 3:** Sensitivity analysis of the efficacy of drugs targeting different species of schistosome (RCTs only)

Study	Method*	No. of Trials	**Participants (N**_**1**_**/N**_**2**_**)**	Pooled RRs (95%CI)	Heterogeneity Test (*P *)
	**A**	**8**	**348/220**	**0.27 (0.22, 0.33)**	**0.88**
PZQ (RCTs) for treatment	B	7	232/177	0.28 (0.22, 0.35)	0.90
	C	6	336/207	0.27 (0.22, 0.32)	0.70
	D	5	220/164	0.28 (0.22, 0.35)	0.70
AM for prevention (8-13 doses by 15-day interval)	**A**	**6**	**2429/2563**	**0.10 (0.05, 0.21)**	**<0.01**
	E	5	1996/2111	0.12 (0.06, 0.24)	<0.01
AS (6 mg/kg) 8 doses by 1-week interval for preventing	**A**	**4**	**813/720**	**0.03 (0.01, 0.12)**	**0.89**
	F	2	572/502	0.02 (0.00, 0.14)	0.73
AS (6 mg/kg) 8-14 doses by 2-week interval for preventing	**A**	**5**	**3317/3553**	**0.05 (0.03, 0.09)**	**0.93**
	G	4	879/893	0.04 (0.01, 0.14)	0.83
AS (6 mg/kg) 3-5 doses by 2-week interval for preventing	**A**	**4**	**714/768**	**0.29 (0.16, 0.52)**	**0.20**
	H	3	334/368	0.39 (0.20, 0.76)	0.39

*A: all trials were included in each subgroup, respectively.

B: one trial [34] with *S. japonicum *species was excluded.

C: two trials [36] with *S. mansoni *species were excluded.

D: three trials [34,36] were excluded, the remaining with *S. haematoium.*

E: one trial [47] in which there was zero person positive in the treatment group was excluded.

F: two trials from the same study [56] were excluded.

G: one trial [53] with the largest sample size was excluded.

H: one trial [52] with the largest sample size was excluded.

### Adverse effects

Adverse effects were reported for people who had taken PZQ for treatment or prevention against schistosomiasis. Among those were dizziness, stomach discomfort or stomach ache, headache, nausea, debility, muscular and joint soreness, and diarrhoea, which disappeared shortly after drug withdrawal [70,71]. In the trials of Tian et al. [42], Song et al. [38,45], Xu et al. [46], Wang et al. [47] and Xiao et al. [48,49] about assessing the efficacy of AM in preventing against *S. japonicum *in the regions of Anhui, Hunan, Jiangxi and Yunnan provinces, only a few of total 4, 754 participants aged from 5 to 60 years old experienced symptoms of mild and transient dizziness, headache, and abdominal pain symptoms with good tolerance and less severe adverse effects [72,73]. AS was also reported being well tolerable. Only about 1% of the participants reported about mild abdominal pain, dizziness, headache and diarrhoea, or slight fever [59].

## Discussion

PZQ was synthesized by Bayer and Merck in Germany in 1972 [74], and introduced for clinical use in the People's Republic of China since 1978 [74,75]. Today, PZQ is the most frequently used drug for schistosomiasis treatment in endemic areas, and regularly used also in large scale programmes [76]. PZQ exhibits stage-specific functions in killing adult worms [73,77,78]. Our meta-analysis showed that a single oral dose of 30-60 mg/kg PZQ or 40/60 mg/kg divided in two doses during RCTs resulted in protection rates of 73% (95% CI: 67%-78%) for treating schistosomiasis. No significant differences were observed among the species *S. haematobium*, *S. japonicum *and *S. mansoni*, but the protective efficacy decreases significantly to 16% (95% CI: -81%-61%) when the PZQ dosage decreased to a single dose of 20 mg/kg. Similarly, 60-100 mg/kg PZQ (i.e. 60 mg/kg as single or double treatment, two doses of 40 mg/kg, or two doses of 30 mg/kg adding a single dose of 40 mg/kg) during nRCTs revealed protection rates of 91% (95% CI: 88%-92%). The protective effect declined significantly to 52% (95% CI: 49%-55%) when the dosage was reduced to a single dose of 40 mg/kg. Thus it can be concluded that multiple doses of 40/60 mg/kg PZQ provide an enhanced efficacy in treating schistosomiasis compared to a single dose. Additionally, when PZQ was applied during the 3^rd^-4^th ^week after the first exposure of a patient to infected water harboring schistosome cercariae, is turned out to be also effective in preventing acute schistosomiasis morbidity with a protections rate of 98% (95% CI: 93%-99%) in two RCTs, and 100% of one nRCT [36,62]. Temporal observations and long-term follow-up investigations about adverse effects showed that PZQ had low toxicity, mild adverse symptoms, no malformation effects, and no sequel. The incidence rates of heavy side-effects were between 0.47% and 1.54% [79]. Thus PZQ has been a safe and effective antischistosomal drug with a broad applicability for all species of schistosome [35,80].

One key issue of chemotherapy for human schistosomiasis treatment is the concern of investigators and clinicians for the emergence of PZQ resistance [81-83]. To date, there is no convincing clinical evidence for schistosome resistance to PZQ used for human schistosomiasis treatment, although worrying low-cure rates have been reported in some studies [84-93]. Our meta-analysis covering a publication period from 1979 to 2009 indicated that PZQ is still effective for *S. haematobium*, *S. mansoni *and *S. japonicum *with negligible variations. We conclude that there is no reason to be worried about the spreading of extensive and serious PZQ resistance, which as King et al. mentioned in 2000 "... will likely take 10 or more years to emerge" [94]. However, one should not lose sight of first evidence for reports indicating the existence of PZQ-tolerable schistosomes which have emerged locally in some African areas [90]. The observed variations between the results of RCTs and nRCTs are explained by the slack design of these nRCTs'. However, dose response trends are the same in RCTs and nRCTs, i.e. larger dosage or more treatment time points increase PZQ's efficacy.

AM and AS, the derivatives of artemisinin (ART), which was first extracted from the sweet wormwood herb *Artemisia annua *by Chinese chemist Youyou Tu and her team in 1970s, were at first used for the treatment of malaria (http://www.laskerfoundation.org/awards/2011_c_description.htm); their potential against *S. japonicum *was first reported in the 1980s [95,96], just some years after the discovery of the antischistosomal properties of artemisinin [97]. ARTs were approved as antischistosomal drugs by the Chinese Ministry of Health in 1990s [74]. AM and AS have similar functions and kill larval worms (schistosomula) of different schistosome species. Thus they have been used as chemoprophylactic drugs against schistosomes and could induce resistance to reinfection [73,74,98]. Our meta-analyses suggest that shortening application intervals could improve their preventive potential. Best efficacies were obtained with 8 or more doses of 6 mg/kg AM applied with 15-day intervals ([90% (95% CI: 79%-95%]), or with 6 mg/kg AS applied with 1-week intervals (97% [95% CI: 88%-99%]). AS, however, showed poor performance with respect to schistosomiasis treatment [24,29] (Table 2). Thus AM and AS seem to be suitable as chemoprophylactic drugs for preventing schistosomiasis morbidity, but they seem not to be competent for treating schistosomiasis. In clinical studies using AM or AS to prevent schistosomiasis incidence rates of less than 1% side-effects were reported [71,74,99,100]. Up to now, there haven't been any reports about drug resistance of schistosomes to these two drugs [101-103] besides one exception. Hua et al [104] reported recently that the sensitivity of artesunate against *S. japonicum *has decreased after 10 years of use in China. The study reported about protection rates obtained after the application of 4 doses of 6 mg/kg AS given over 3-day intervals between the 1^st ^and the 2^nd ^dose and the 3^rd ^and the 4^th ^dose, A 11-day interval occurred between the 2^nd ^and the 3^rd ^treatment. Surprisingly, the protection rate during that time period was 74.8%, but it fell to 13.5% when the AS application was changed to 4 doses of 6 mg/kg AS given in 1-week intervals. This unexpected result is difficult to reconcile with similar study reports, and because of its not-perfect study design and contradictory statements between study design and drug administration of participants, why it was not included in our meta-analysis. Although the authors concluded that they identified potential artemisinin-derivate-resistance in *S. japonicum*, we believe, that more rigorous research is needed to confirm this finding [105].

Our meta-analysis showed that PZQ and AS in combination provided similar preventive efficacy than those obtained by AM or AS administrated as single drugs (approximately 90%-97%). In conclusion, AM or AS alone have the capacity as prophylactic drugs for schistosomiasis prevention. PZQ and AM or AS in combination for treatment resulted in higher protection rates (84% [95% CI: 64%-91%]) than PZQ alone (73% [95% CI: 67%-78%]), respectively. Thus PZQ-ARTs combinations are useful for schistosomiasis patients repeatedly exposed to infected water. It's well known that ARTs are used in large-scale programmes for malaria treatment (mostly epidemic in Africa). However, ARTs-resistant malarial parasites have been identified and confirmed in some areas [106-108]. Thus using ARTs for schistosomiasis control is not advisable in areas where schistosomiasis and malaria are co-endemic.

One limitation of this meta-analysis is that the diagnostic approaches used to determine parasites were different among the included trials. There were differences between Kato-Katz technique and urine filtration, the number of specimens, and the number of examinations between trials. The former cannot be changed because it is determined by the two kinds of different clinical pathogenesis. The latter can be improved through using a standardized, quality-controlled diagnostic criterion within and between trials. As discussed by Bergquist et al. [109] and Danso-Appiah et al. [110], different diagnostic methods own different sensitivity and specificity, and the same method also owns different sensitivity and specificity for different infection levels and different numbers of specimens, which were called the diagnostic dilemmas. In the current meta-analyses, we tried to stratify the included trials based on their diagnostic approaches and the numbers of specimens and examinations. However, we were restricted doing this since many of the included trials didn't report about the numbers of specimens and examinations for each participant. Thus a unified study design is urgently needed to make the study outcomes more comparable.

The results of the RCTs dealing with PZQ or ART efficacies (using 40 mg/kg for treatment; 8-13 doses of 6 mg/kg AM with 15-day intervals for preventing, and AS for treatment) have *P *values <0.05, which indicates that the studies included in these three subgroups were heterogeneous. We presume that some factors such as e.g. regional variances, participants' differences, or drug dosage differences among these studies, may have contributed to this heterogeneity [111]. RCTs are preferable to be conducted when assessing drug efficacies in order to exclude the influence of such variable factors. Different species of schistosome were thought to have different susceptibility to these drugs, and we stratified the included trials and performed subgroup meta-analysis. Interestingly, the results provided no hint in this direction when different species were used in RCTs using PZQ (30-60 mg/kg for schistosomiasis treatment), AM (6-7 doses of using 6 mg/kg in 1-month intervals for preventing schistosomiasis), and PZQ + ART combinatory schistosomiasis treatment, in which the three schistosome species *S. haematobium*, *S. japonicum *and *S. mansoni *were studied (Figures 2, 3 and 5). We conclude that these drugs may be equally effective against these schistosome species, which we confirmed also by the results of sensitivity analysis (Table 3).

Publication bias is usually cited as a reason for the lack of validity in meta-analyses [112]. Publication bias can occur when studies that found no significant effect of antischistosomal drugs in schistosomiasis control are less likely submitted and accepted for publication than those with a positive result. The funnel plots of the included trials showed no evidence for publication bias except the subgroup of 8-13 doses of at 6 mg/kg AM given with 15-day intervals for preventing schistosomiasis. Two points (represented two trials) were outside the cutoff line, and the *P *value of Egger's publication bias test was 0.01, implying the existence of publication bias.

Additionally, we had found two Cochrane reviews discussing drugs for human schistosomiasis treatment [113,114], one review examined PZQ, metrifonate and artemisinin derivatives used alone or in combination for treating urinary schistosomiasis, and the other assessed the effects of oxamniquine and praziquantel on treatment against *S. mansoni*. Thus our meta-analysis represents a good complementary data set adding to the results obtained in these studies.

## Conclusions

Facing the fact that an integrated strategy, which emphasizes health education, access to clean water and adequate sanitation, mechanization of agriculture, and fencing of water buffaloes (mainly for schistosomiasis japonica endemic areas), along with mass chemotherapy for both human and livestock, have been suggested to be carried out in parallel to control the infection sources and to stop schistosome transmission [115-118], we strived to conduct this meta-analysis hoping to provide a scientific basis for monitoring and selecting antischistosomal drugs efficiently, which is a crucial job for the schistosomiasis integrated control programme. With respect to the results obtained in our meta-analyses, we recommend to use multiple doses of AM or AS at 6 mg/kg given in 1- or 2-week intervals for prevention against schistosome infection during exposure to infected water. PZQ remains effective in schistosomiasis treatment, and multiple doses of PZQ at 30-60 mg/kg can improve its efficacy. PZQ and AM or AS administrated in combination are more effective than single drug PZQ therapy, which maybe especially suitable for the treatment of patients, who are repeatedly exposed to infected water.

## List of abbreviations used

AM: artemether; ARTs: artemisinin derivatives; AS: artesunate; CBM: Chinese Biomedicine Literature Database; CNKI: China National Knowledge Infrastructure; DALYs: disability-adjusted life years; nRCT(s): non-randomized controlled trial(s); PZQ: praziquantel; P: prevention; RCT(s): randomized controlled trial(s); RR(s): relative risk(s); *Sh: Schistosoma haematobium; Sj: Schistosoma japonicum; *T: treatment; yr: years old; 95% CI(s): 95 percent confidence interval(s).

## Competing interests

The authors declare that they have no competing interests.

## Authors' contributions

RL, HFD and MSJ designed this work. RL and YG searched databases, selected studies and abstracted information. RL entered and analyzed data, drafted the manuscript. RL, HFD and YG contributed to the interpretation of results. RL, HFD, MSJ and QPZ contributed to the refinement of the manuscript. RL, HFD and MSJ revised the manuscript. All of authors have read and approved the final version of the manuscript.

## Supplementary Material

Additional file 1 **Table S1 Summary of the characteristics of the included trials in our meta-analyses evaluating antischistosomal drugs (PZQ, AM and AS), used alone or in combination, for human schistosomiasis treatment or prevention**. This table describes the extracted characteristics of the included studies: Author, Year; Test sites; Time; Participants; Species; Interventions; Diagnostic approach; Follow-up time; Treated (n/N); Controlled (n/N); Relative Risk; (95%CI); Design Type; Usage. Click here for file

## References

[B1] Chitsulo L Engels D Montresor A Savioli L The global status of schistosomiasis and its control Acta Trop 2000 77 41 51 10.1016/S0001-706X(00)00122-4 10996119 PMC5633072

[B2] King CH Dickman K Tisch DJ Reassessment of the cost of chronic helminthic infection: a meta-analysis of disability-related outcomes in endemic schistosomiasis Lancet 2005 365 1561 1569 10.1016/S0140-6736(05)66457-4 15866310

[B3] Gryseels B Polman K Clerinx J Kestens L Human schistosomiasis Lancet 2006 368 1106 1118 10.1016/S0140-6736(06)69440-3 16997665

[B4] Utzinger J Raso G Brooker S De Savigny D Tanner M Ornbjerg N Singer BH N'goran EK Schistosomiasis and neglected tropical diseases: towards integrated and sustainable control and a word of caution Parasitology 2009 136 1859 1874 10.1017/S0031182009991600 19906318 PMC2791839

[B5] Mathers CD Ezzati M Lopez AD Measuring the burden of neglected tropical diseases: the global burden of disease framework PLoS Negl Trop Dis 2007 1 e114 10.1371/journal.pntd.0000114 18060077 PMC2100367

[B6] Hotez PJ Molyneux DH Fenwick A Kumaresan J Sachs SE Sachs JD Savioli L Control of neglected tropical diseases N Engl J Med 2007 357 1018 1027 10.1056/NEJMra064142 17804846

[B7] Hotez PJ Brindley PJ Bethony JM King CH Pearce EJ Jacobson J Helminth infections: the great neglected tropical diseases J Clin Invest 2008 118 1311 1321 10.1172/JCI34261 18382743 PMC2276811

[B8] Ross AG Bartley PB Sleigh AC Olds GR Li Y Williams GM McManus DP Schistosomiasis N Engl J Med 2002 346 1212 1220 10.1056/NEJMra012396 11961151

[B9] McManus DP Loukas A Current status of vaccines for schistosomiasis Clin Microbiol Rev 2008 21 225 242 10.1128/CMR.00046-07 18202444 PMC2223839

[B10] WHO/SCHISTO The role of chemotherapy in schistosomiasis control Geneva 1983 WHO 70

[B11] Doenhoff MJ Cioli D Utzinger J Praziquantel: mechanisms of action, resistance and new derivatives for schistosomiasis Curr Opin Infect Dis 2008 21 659 667 10.1097/QCO.0b013e328318978f 18978535

[B12] Botros S Sayed H Amer N El-Ghannam M Bennett JL Day TA Current status of sensitivity to praziquantel in a focus of potential drug resistance in Egypt Int J Parasitol 2005 35 787 791 10.1016/j.ijpara.2005.02.005 15925597

[B13] King CH Muchiri EM Ouma JH Evidence against rapid emergence of praziquantel resistance in *S. haematobium*, Kenya Emerg Infect Dis 2000 6 585 594 10.3201/eid0606.000606 11076716 PMC2640915

[B14] Silva IM Thiengo R Conceição MJ Rey L Lenzi HL Pereira Filho E Ribeiro PC Therapeutic failure of praziquantel in the treatment of *Schistosoma haematobium *infection in Brazilians returning from Africa Mem Inst Oswaldo Cruz 2005 100 445 449 16113896 10.1590/s0074-02762005000400018

[B15] Alonso D Muñoz J Gascón J Valls ME Corachan M Failure of standard treatment with praziquantel in two returned travellers with *Schistosoma haematobium *infection Am J Trop Med Hyg 2006 74 342 344 16474094

[B16] Wang ZH Research progress of schistosomiasis japonica chemotherapy Hubei J Prev Med 2000 11 1 3 (in Chinese)

[B17] Xiao SH Catto BA In vitro and in vivo studies of the effect of artemether on *Schistosoma mansoni* Antimicrob Agents Chemother 1989 33 1557 1562 2510595 10.1128/aac.33.9.1557 PMC172702

[B18] Xiao SH You JQ Yang YQ Wang CZ Experimental studies on early treatment of schistosomal infection with artemether Southeast Asian J Trop Med Public Health 1995 26 306 318 8629066

[B19] Xiao SH Utzinger J Chollet J Endriss Y N'Goran EK Tanner M Effect of artemether against *Schistosoma haematobium *in experimentally infected hamsters Int J Parasitol 2000 30 1001 1006 10.1016/S0020-7519(00)00091-6 10980290

[B20] Utzinger J Keiser J Xiao SH Tanner M Singer BH Combination chemotherapy of schistosomiasis in laboratory studies and clinical trials Antimicrob Agents Chemother 2003 47 1487 1495 10.1128/AAC.47.5.1487-1495.2003 12709312 PMC153321

[B21] Xiao SH Tanner M N'Goran EK Utzinger J Chollet J Bergquist R Chen MG Zheng J Recent investigations of artemether, a novel agent for the prevention of schistosomiasis japonica, mansoni and haematobia Acta Trop 2002 82 175 181 10.1016/S0001-706X(02)00009-8 12020890

[B22] Higgins JPT Green S (editors) Cochrane Handbook for Systematic Reviews of Interventions Version 4.2.6 [updated September 2006] The Cochrane Collaboration 2006 http://www.cochrane.org/resources/handbook/

[B23] Jadad AR Moore RA Carroll D Jenkinson C Reynolds DJ Gavaghan DJ McQuay HJ Assessing the quality of reports of randomized clinical trials: is blinding necessary? Control Clin Trials 1996 17 1 12 10.1016/0197-2456(95)00134-4 8721797

[B24] Inyang-Etoh PC Ejezie GC Useh MF Inyang-Etoh EC Efficacy of a combination of praziquantel and artesunate in the treatment of urinary schistosomiasis in Nigeria Trans R Soc Trop Med Hyg 2009 103 38 44 10.1016/j.trstmh.2008.08.002 18838149

[B25] Touré S Zhang Y Bosqué-Oliva E Ky C Ouedraogo A Koukounari A Gabrielli AF Bertrand S Webster JP Fenwick A Two-year impact of single praziquantel treatment on infection in the national control programme on schistosomiasis in Burkina Faso Bull World Health Organ 2008 86 780 787 10.2471/BLT.07.048694 18949215 PMC2649514

[B26] Tohon ZB Mainassara HB Garba A Mahamane AE Bosqué-Oliva E Ibrahim ML Duchemin JB Chanteau S Boisier P Controlling schistosomiasis: significant decrease of anaemia prevalence one year after a single dose of praziquantel in Nigerien schoolchildren PLoS Negl Trop Dis 2008 2 e241 248 10.1371/journal.pntd.0000241 18509472 PMC2386241

[B27] Saathoff E Olsen A Magnussen P Kvalsvig JD Becker W Appleton CC Patterns of *Schistosoma haematobium *infection, impact of praziquantel treatment and re-infection after treatment in a cohort of schoolchildren from rural KwaZulu-Natal/South Africa BMC Infect Dis 2004 4 40 49 10.1186/1471-2334-4-40 15471549 PMC524490

[B28] N'Goran EK Gnaka HN Tanner M Utzinger J Efficacy and side-effects of two praziquantel treatments against *Schistosoma haematobium *infection, among school children from Côte d'Ivoire Ann Trop Med Parasitol 2003 97 37 51 10.1179/000349803125002553 12662421

[B29] Borrmann S Szlezák N Faucher JF Matsiegui PB Neubauer R Binder RK Lell B Kremsner PG Artesunate and praziquantel for the treatment of *Schistosoma haematobium *infections: a double-blind, randomized, placebo-controlled study J Infect Dis 2001 184 1363 1366 10.1086/324004 11679932

[B30] Campagne G Garba A Barkiré H Vera C Sidiki A Chippaux JP Continued ultrasonic follow-up of children infected with *Schistosoma haematobium *after treatment with praziquantel Trop Med Int Health 2001 6 24 30 10.1046/j.1365-3156.2001.00660.x 11263461

[B31] Utzinger J N'Goran EK N'Dri A Lengeler C Tanner M Efficacy of praziquantel against *Schistosoma mansoni *with particular consideration for intensity of infection Trop Med Int Health 2000 5 771 778 10.1046/j.1365-3156.2000.00646.x 11123824

[B32] Nash TE Hofstetter M Cheever AW Ottesen EA Treatment of *Schistosoma mekongi *with praziquantel: a double-blind study Am J Trop Med Hyg 1982 31 977 982 6751116 10.4269/ajtmh.1982.31.977

[B33] Santos AT Blas BL Noseñas JS Portillo GP Ortega OM Hayashi M Boehme K Preliminary clinical trials with praziquantel in *Schistosoma japonicum *infections in the Philippines Bull World Health Organ 1979 57 793 799 396056 PMC2395875

[B34] McMahon JE Kolstrup N Praziquantel: a new schistosomicide against *Schistosoma haematobium* Br Med J 1979 2 1396 1399 10.1136/bmj.2.6202.1396 519476 PMC1597073

[B35] Katz N Rocha RS Chaves A Preliminary trials with praziquantel in human infections due to *Schistosoma mansoni* Bull World Health Organ 1979 57 781 785 396054 PMC2395863

[B36] Huang YX Rong GR Xu GY Wang XD Yang HM Song HT Zhang XB Tu YX Mass praziquantel chemoprophylaxis against acute schistosomiasis japonica in a flood Chin J Schisto Control 1998 10 138 140 (in Chinese)

[B37] Liu ZG Effect of intermittent medication with praziquantel for treating schistosomiasis japonica in the endemic seasons Chin J Parasit Dis Control 1997 10 21 (in Chinese)

[B38] Song Y Bao ZP Gao ZL Ning A Hu QL Chen MG Chen FJ Ge J Xiao SH Zhou XN Xu J Effect of oral artemether in controlling schistosomiasis in a heavy endemic area of Nanji town, Xinjian county, Jiangxi province J Trop Med 2006 6 1182 1185 (in Chinese)

[B39] Li YS Chen HG He HB Hou XY Ellis M McManus DP A double-blind field trial on the effects of artemether on *Schistosoma japonicum *infection in a highly endemic focus in southern China Acta Trop 2005 96 184 190 10.1016/j.actatropica.2005.07.013 16112071

[B40] N'Goran EK Utzinger J Gnaka HN Yapi A N'Guessan NA Kigbafori SD Lengeler C Chollet J Xiao SH Tanner M Randomized, double-blind, placebo-control trial of oral artemether for the prevention of patent *Schistosoma haematobium *infections Am J Trop Med Hyg 2003 68 24 32 12556143

[B41] Utzinger J N'Goran EK N'Dri A Tanner M Oral artemether for prevention of *Schistosoma mansoni *infection: randomized controlled trial Lancet 2000 355 1320 1325 10.1016/S0140-6736(00)02114-0 10776745

[B42] Tian ZY Xiao SH Xiao JW Liu DS Zhou YC Zheng J Chen MG Qu GS Zhang XY Yao XM Zhang XZ Zhang DL Huang GX Reduction of *Schistosoma japonicum *infection in an endemic area in Islet with embankment after prophylaxis with oral artemether throughout the transmission season Chin J Parasito Parasitic Dis 1997 15 208 211 (in Chinese)

[B43] Tian ZY Liu DS Xiao JW Yao XM Zhou YC Qu GS Zhang XR Efficacy investigation of fishermen taking oral artemether on schistosomiasis prevention in Muping Lake Chin J Schisto Control 1999 11 317 (in Chinese)

[B44] Huang AS Zhou B Chen WY Sheng QH Jing GY Xiao CY Xiao SH Chen MG Zheng J Liu HX Preliminary investigation of effect of artemether in people working with water on schistosomiasis prevention Chin J Schisto Control 1999 11 161 (in Chinese)

[B45] Song Y Xiao S Wu W Zhang S Xie H Xu X Hu X Cui Q Chen M Zheng J Preventive effect of artemether on schistosome infection Chin Med J (Engl) 1998 111 123 127 10374370

[B46] Xu MS Xiao SH Song Q Tao CG Xia CG Wang H Chen MG Zheng J Piao CH Hu FY Ou N Zhang XS Observation on the effect of artemether on controlling schistosomiasis japonica in an endemic area of marshland Chin J Parasito Parasitic Dis 1997 15 212 215 (in Chinese)

[B47] Wang JL Xiao SH Yang Z Huang MH Yang H Liu YH Zhou GS Zheng J Chen MG Effect of oral artemether in controlling schistosomiasis in Yunnan mountainous endemic Area Chin J Parasitol Parasitic Dis 1997 15 138 142 (in Chinese)

[B48] Xiao SH Shi ZG Zhuo SJ Wang CZ Zhang ZG Chu B Zheng J Chen MG Field studies on preventive effect of artemether against infection with *Schistosoma japonicum* Chin J Parasitol Parasitic Dis 1995 13 170 173 (in Chinese) 8556789

[B49] Xiao SH Wang JL Wang CZ Yang Z Chu B Yang H Liu YH Zheng J Chen MG Protection of the residents from schistosome infection by oral artemether in mountainous endemic area Chin J Parasitol Parasitic Dis 1996 14 11 14 (in Chinese)

[B50] Xu MS Zhu CG Wang H Gao FH Wu YX Cui DY Zhang XZ Ou N Wu ZX Field study on preventive effect of artesunate against infection due to *Schistosoma japonicum *in an endemic area of marshlands J Trop Dis Parasitol 1996 25 198 201 (in Chinese)

[B51] Xu MS Zhang SQ Wang TP Fang GR Wang QZ He JC Zhang GH Chen JR Li JT Lu YS Li XS Xu CS Bao JR Yu MX Huang FY Ou N Zhang XS Piao CH Hu FY Cui DY Field appliance of oral artesunate for prevention of schistosomiasis japonica J Trop Dis Parasitol 1998 7 68 71 (in Chinese)

[B52] Xu MS The effect analysis of using artesunate to prevent schistosome infection in the field Chin J Parasit Dis Control 2001 14 26 27 (in Chinese)

[B53] Cui JF Lu GY Lin GJ Sun MX Jiang J Wu QZ Li JS Study of artesunate in preventing schistosome infection in river beach area Anhui Prev Med 2001 7 129 130 (in Chinese)

[B54] Sun MX Jiang J Lu GY Lin GJ Cui JF Wu QZ Li JS Field investigation of oral artesunate for preventing high risk population from schistosome infection Anhui J Prev Med 2000 6 442 (in Chinese)

[B55] Zhang SJ Lin DD Liu YM Liu HY Liu ZD Hu LS Gao ZL Hu F Xu MS Yi ZH Wu LJ Li SW Clinical trials on preventive effect of artesunate on schistosomiasis japonica Mod Diagn Treat 2000 11 68 72 (in Chinese)

[B56] Yi ZH Lu M Feng DC Wang ZH Xiang CP Gou ZQ Li SW Wu LJ Clinical observation of oral artesunate for preventing short-term exposure to infested water population from schistosomiasis Chin J Schisto Control 2000 12 100 101 (in Chinese)

[B57] Liu HY Liu ZD Hu LS Liu YM Zhang SJ Hu F Cheng XY Li SW Wu LJ Xu FS Xuan YX Guo Y Observation on the prevention of schistosomiasis japonica by administration of artesunate long term Chin J Parasitic Dis Control 1999 12 214 215 (in Chinese)

[B58] Liu ZD Hu LS Liu YM Hu GH Hu F Qiu YX Gao ZL Liu HY Li JY Su LH Expanded experimental study on the prevention of schistosomiasis japonica by oral artesunate Chin J Parasitic Dis Control 1996 9 37 39 (in Chinese)

[B59] Wu LJ Li SW Xuan YX Xu PS Liu ZD Hu LS Zhou SY Qiu YX Liu YM Field application of artesunate in prophylaxis of schistosomiasis: an observation of 346 cases Chin J Schisto Control 1995 7 323 327 (in Chinese)

[B60] Hou XY McManus DP Gray DJ Balen J Luo XS He YK Ellis M Williams GM Li YS A randomized, double-blind, placebo-controlled trial of safety and efficacy of combined praziquantel and artemether treatment for acute schistosomiasis japonica in China Bull World Health Organ 2008 86 788 795 10.2471/BLT.08.053041 18949216 PMC2649525

[B61] Xia CS Zhao XY Liu YF Jiang ZH Li NP Observation of the efficacy on schistosomiasis prevention with artesunate and praziquantel J Prev Med Chin PLA 2000 18 120 121 (in Chinese)

[B62] Liu ZG Yuan XB Efficacy investigation of praziquantel in schistosomiasis prevention Hubei J Prev Med 1997 8 30 31 (in Chinese)

[B63] Song Y Xiao SH Wu W Zhang SJ Xie HQ Xu XP Hu XY Cui Q Chen MG Zheng J The preventive effect of artemether in protection of people from schistosome infection during fighting against flood Chin J Parasitol Parasitic Dis 1997 15 133 137 (in Chinese)

[B64] Chen HG Lin DD Li YS Liu YM McManus DP Huang XH Feng Z Studies on effect of artemether to control infection and prevent acute infection of *Schistosoma japonicum *in high endemic areas Chin J Schisto Control 2006 18 32 35 (in Chinese)

[B65] Xu MS Zhu CG Wang H Gao FH Wu YX Cui DY Zhang XZ Ou N Wu ZX Study on preventive effect of artesunate against infection due to *Schistosoma japonicum *in an endemic area of marshlands Chin J Schisto Control 1997 9 268 271 (in Chinese)

[B66] Hou XY Li YS Luo XS He YK Yu XL Fu X Zhou J Shi MZ Liu ZC Wang YY Li Y Wei WY Clinical research of combination praziquantel and artemether in treating acute schistosomiasis japonica Chin J Schisto Control 2006 18 99 102 (in Chinese)

[B67] Huang YX Wang WF Xue ZQ Yang SJ Chen YQ Wu YX Zhou JX Double blind investigation of praziquantel in treating schistosomiasis japonica Jiangsu Med Drug 1983 1 19 21 (in Chinese)

[B68] Olliaro PL Vaillant MT Belizario VJ Lwambo NJ Ouldabdallahi M Pieri OS Amarillo ML Kaatano GM Diaw M Domingues AC Favre TC Lapujade O Alves F Chitsulo L A multi-centre randomized controlled trial of the efficacy and safety of single-dose praziquantel at 40 mg/kg vs. 60 mg/kg for treating intestinal schistosomiasis in the Philippines, Mauritania, Tanzania and Brazil PLoS Negl Trop Dis 2011 5 e1165 10.1371/journal.pntd.0001165 21695161 PMC3114749

[B69] Hua HY Liang YS Zhang Y Wei JF Guo HX The sensitivity of artesunate against *Schistosoma japonicum *decreased after 10 years of use in China Parasitol Res 2010 107 873 878 10.1007/s00436-010-1944-5 20549236

[B70] Zhou ZB Li WT Xiong SL Xu YB Li YB Lan YH Zhao CG Rao CD Mao AJ Liu SQ Du GY Li Y Clinical analysis of praziquantel in treating schistosomiasis japonica: 1107 cases report Yunnan Med Drug 1983 4 148 152 (in Chinese)

[B71] Utzinger J Zhou XN Chen MG Bergquist R Conquering schistosomiasis in China: the long march Acta Trop 2005 96 69 96 16312039 10.1016/j.actatropica.2005.08.004

[B72] Wang ZH Chemical treatment of schistosomiasis japonica in China J Clin Intern Med 2006 23 302 304 (in Chinese)

[B73] Xiao SH Study on prevention and cure of artemether against schistosomiasis Chin J Schisto Control 2005 17 310 320 (in Chinese)

[B74] Xiao SH Development of antischistosomal drugs in China, with particular consideration to praziquantel and the artemisinins Acta Trop 2005 96 153 167 10.1016/j.actatropica.2005.07.010 16112072

[B75] Midzi N Sangweme D Zinyowera S Mapingure MP Brouwer KC Kumar N Mutapi F Woelk G Mduluza T Efficacy and side effects of praziquantel treatment against *Schistosoma haematobium *infection among primary school children in Zimbabwe Trans R Soc Trop Med Hyg 2008 102 759 766 10.1016/j.trstmh.2008.03.010 18486169

[B76] Ma YJ Guo M Liu JF Chemotherapeutic drugs against schistosomiasis japonica Endemic Dis Bull 2007 22 68 69 (in Chinese)

[B77] Xiao SH Yue WJ Yang YQ You JQ Susceptibility of *Schistosoma japonicum *to different developmental stages to praziquantel Chin Med J (Engl) 1987 100 759 768 3127152

[B78] Keiser J Chollet J Xiao SH Mei JY Jiao PY Utzinger J Tanner M Mefloquine--an aminoalcohol with promising antischistosomal properties in mice PLoS Negl Trop Dis 2009 3 e350 10.1371/journal.pntd.0000350 19125172 PMC2600813

[B79] Wu WL Huang YY Zhou XZ Wang JX Zhang SY Zhang YM Lu JY He WB Lang YG Tang HJ Yang SG A 19-year follow-up study on praziquantel treatment for schistosomiasis Chin J Schisto Control 1998 10 215 217 (in Chinese)

[B80] Adam I Elwasila E Homeida M Praziquantel for the treatment of schistosomiasis mansoni during pregnancy Ann Trop Med Parasitol 2005 99 37 40 10.1179/136485905X17407 15701253

[B81] Fallon PG Tao LF Ismail MM Bennett JL Schistosome resistance to praziquantel: Fact or artifact? Parasitol Today 1996 12 316 320 10.1016/0169-4758(96)10029-6 15275183

[B82] Fallon PG Schistosome resistance to praziquantel Drug Resist Updat 1998 1 236 241 10.1016/S1368-7646(98)80004-6 16904406

[B83] Seto EY Wong BK Lu D Zhong B Human schistosomiasis resistance to praziquantel in china: should we be worried? Am J Trop Med Hyg 2011 85 74 82 10.4269/ajtmh.2011.10-0542 21734129 PMC3122348

[B84] Shi MZ Yu DB Wei WY Zhang CS He HB Yang GF Li GP Ren MY Experimental study on susceptibility of praziquantel against *Schistosoma japonicum *in repeated chemotherapy areas in Dongting Lake region Chin J Schisto Contr 2004 16 171 173 (in Chinese)

[B85] Doenhoff MJ Pica-Mattoccia L Praziquantel for the treatment of schistosomiasis: its use for control in areas with endemic disease and prospects for drug resistance Expert Rev Anti Infect Ther 2006 4 199 210 10.1586/14787210.4.2.199 16597202

[B86] Danso-Appiah A de Vlas SJ Interpreting low praziquantel cure rates of *Schistosoma mansoni *infections in Senegal Trends Parasitol 2002 18 125 129 10.1016/S1471-4922(01)02209-7 11854090

[B87] Ismail M Metwally A Farghaly A Bruce J Tao LF Bennett JL Characterization of isolates of *Schistosoma mansoni *from Egyptian villagers that tolerate high doses of praziquantel Am J Trop Med Hyg 1996 2 214 218 10.4269/ajtmh.1996.55.214 8780463

[B88] Liang YS Coles GC Doenhoff MJ Southgate VR In vitro responses of praziquantel-resistant and -susceptible *Schistosoma mansoni *to praziquantel Int J Parasitol 2001 11 1227 1235 10.1016/s0020-7519(01)00246-6 11513892

[B89] Kenworthy JD Ye P Wu GC Yu H Shi YJ Li H Coles GC Field evaluation of a test for praziquantel resistance in *Schistosoma *sp Vet Parasitol 2003 1 83 87 10.1016/s0304-4017(03)00036-0 12651219

[B90] Ismail M Botros S Metwally A William S Farghally A Tao LF Day TA Bennett JL Resistance to praziquantel: direct evidence from *Schistosoma mansoni *isolated from Egyptian villagers Am J Trop Med Hyg 1999 60 932 395 10403323 10.4269/ajtmh.1999.60.932

[B91] Gryseels B Mbaye A de Vlas SJ Stelma FF Guissé F Van Lieshout L Faye D Diop M Ly A Tchuem-Tchuenté LA Engels D Polman K Are poor responses to praziquantel for the treatment of *Schistosoma mansoni *infections in Senegal due to resistance? An overview of the evidence Trop Med Int Health 2001 6 864 873 10.1046/j.1365-3156.2001.00811.x 11703840

[B92] King CH Muchiri EM Ouma JH Evidence against rapid emergence of praziquantel resistance in *Schistosoma haematobium*, Kenya Emerg Infect Dis 2000 6 585 594 10.3201/eid0606.000606 11076716 PMC2640915

[B93] Kasinathan RS Morgan WM Greenberg RM *Schistosoma mansoni *express higher levels of multidrug resistance-associated protein 1 (SmMRP1) in juvenile worms and in response to praziquantel Mol Biochem Parasitol 2010 173 25 31 10.1016/j.molbiopara.2010.05.003 20470831 PMC2896741

[B94] Doenhoff MJ Hagan P Cioli D Southgate V Pica-Mattoccia L Botros S Coles G Tchuem Tchuenté LA Mbaye A Engels D Praziquantel: its use in control of schistosomiasis in sub-Saharan Africa and current research needs Parasitology 2009 136 1825 1835 10.1017/S0031182009000493 19281637

[B95] Le WJ You JQ Yang YQ Mei JY Guo HF Yang HZ Zhang CW Studies on the efficacy of artemether in experimental schistosomiasis Acta Pharm Sin 1982 17 187 193 7115549

[B96] Le WJ You JQ Mei JY Chemotherapeutic effect of artesunate in experimental schistosomiasis Acta Pharm Sin 1983 18 619 621 6677044

[B97] Chen DJ Fu LF Shao PP Wu FZ Shu H Ren CS Sheng XL Experimental studies on antischistosomal activity of qinghaosu Zhong Hua Yi Xue Zha Zhi 1980 60 422 425

[B98] Bartley PB Glanfield A Li YS Stanisic DI Duke M Jones MK McManus DP Artemether treatment of prepatent *Schistosoma japonicum *induces resistance to reinfection in association with reduced pathology Am J Trop Med Hyg 2008 78 929 935 18541772 PMC2756499

[B99] Utzinger J Xiao SH N'Goran EK Bergquist R Tanner M The potential of artemether for the control of schistosomiasis Int J Parasitol 2001 31 1549 1562 10.1016/S0020-7519(01)00297-1 11730781

[B100] Zhou J Wu GH Development of the drug to prevent schistosomiasis japonica Chin J Dis Control Prev 2002 6 71 73 (in Chinese)

[B101] Xiao SH Study on prevention and cure of artemether against schistosomiasis Chin J Schisto Control 2005 17 310 320 (in Chinese)

[B102] Division of Science and Education of Chinese Ministry of Health Research on artemether and artesunate for prevention of schistosomiasis China Medical News 1999 13 6 8 (in Chinese)

[B103] Xu MS Research progress of artemisinin, artemether and artesunate used for schistosomiasis prevention Chin J Schisto Control 1998 4 251 253 (in Chinese)

[B104] Hua HY Liang YS Zhang Y Wei JF Guo HX The sensitivity of artesunate against *Schistosoma japonicum *decreased after 10 years of use in China Parasitol Res 2010 107 873 878 10.1007/s00436-010-1944-5 20549236

[B105] Liu R Dong HF Jiang MS The sensitivity of artesunate against *Schistosoma japonicum *decreased after 10 years of use in China? Parasitol Res 2011 10.1007/s00436-011-2641-8 21912960

[B106] Dondorp AM Nosten F Yi P Das D Phyo AP Tarning J Lwin KM Ariey F Hanpithakpong W Lee SJ Ringwald P Silamut K Imwong M Chotivanich K Lim P Herdman T An SS Yeung S Singhasivanon P Day NP Lindegardh N Socheat D White NJ Artemisinin resistance in *Plasmodium falciparum malaria* N Engl J Med 2009 361 455 467 10.1056/NEJMoa0808859 19641202 PMC3495232

[B107] Editorial Has artemisinin resistance spread already? Lancet 2011 377 272 21256369 10.1016/S0140-6736(11)60077-9

[B108] Cui W WHO urges the phasing out of artemisinin based monotherapy for malaria to reduce resistance BMJ 2011 342 d2793 10.1136/bmj.d2793 21543403

[B109] Bergquist R Johansen MV Utzinger J Diagnostic dilemmas in helminthology: what tools to use and when? Trends Parasitol 2009 25 151 156 10.1016/j.pt.2009.01.004 19269899

[B110] Danso-Appiah A Garner P Olliaro PL Utzinger J Treatment of urinary schistosomiasis: methodological issues and research needs identified through a Cochrane systematic review Parasitology 2009 136 1837 1849 10.1017/S0031182009005939 19493363

[B111] Liu SJ Cao HN Tao B Li QR Cheng LG Wu ZX Xiong TS Deng LX Chen LH Zuo XX Song GX Zhang R Efficacy investigation of praziquantel in treating different infection level schistosomiasis Chin J Parasitol Parasitic Dis 1989 7 64 65 (in Chinese)

[B112] Li YP (editor) Evidence-based medicine 2003 Beijing: Higher Education Press (in Chinese)

[B113] Danso-Appiah A Utzinger J Liu J Olliaro P Drugs for treating urinary schistosomiasis Cochrane Database Syst Rev 2008 3 CD000053 18646057 10.1002/14651858.CD000053.pub2

[B114] Saconato H Atallah A Interventions for treating schistosomiasis mansoni Cochrane Database Syst Rev 2000 2 CD000528 10.1002/14651858.CD000528 10796552

[B115] Wang LD Utzinger J Zhou XN Schistosomiasis control: experiences and lessons from China Lancet 2008 372 1793 1795 10.1016/S0140-6736(08)61358-6 18930529 PMC7135384

[B116] Wang LD Guo JG Wu XH Chen HG Wang TP Zhu SP Zhang ZH Steinmann P Yang GJ Wang SP Wu ZD Wang LY Hao Y Bergquist R Utzinger J Zhou XN China's new strategy to block *Schistosoma japonicum *transmission: experiences and impact beyond schistosomiasis Trop Med Int Health 2009 14 1475 1483 10.1111/j.1365-3156.2009.02403.x 19793080

[B117] Jiang MS Liu R Zhao QP Dong HF Guo Y Social epidemiological thinking about schistosomiasis Chin J Schisto Contr 2010 22 201 205 (in Chinese)

[B118] Gray DJ McManus DP Li YS Williams GM Bergquist R Ross AG Schistosomiasis elimination: lessons from the past guide the future Lancet Infect Dis 2010 10 733 736 10.1016/S1473-3099(10)70099-2 20705513

