# Supplementary material for: Efficacy of praziquantel and artemisinin derivatives for the treatment and prevention of human schistosomiasis: a systematic review and meta-analysis
Source: Parasit Vectors. 2011 Oct 17;4:201. doi: 10.1186/1756-3305-4-201 (PMC3207908; doi:10.1186/1756-3305-4-201)
Supplement: Additional file 1 — Table S1 Summary of the characteristics of the included trials in our meta-analyses evaluating antischistosomal drugs (PZQ, AM and AS), used alone or in combination, for human schistosomiasis treatment or prevention. This table describes the extracted characteristics of the included studies: Author, Year; Test sites; Time; Participants; Species; Interventions; Diagnostic approach; Follow-up time; Treated (n/N); Controlled (n/N); Relative Risk; (95%CI); Design Type; Usage. [file 1756-3305-4-201-S1.DOC]

Table S1 Summary of the characteristics of the included trials in our meta-analyses evaluating antischistosomal drugs (PZQ, AM and AS), used alone or in combination, for human schistosomiasis treatment or prevention

| **Author, Year** | **Test sites** | **Time** | **Participants** | **Species** | **Interventions** | **Diagnostic approach** | **Follow-up time** | **Treated (n/ N^△^)** | **Controlled (n/ N)** | **Relative Risk**  **(95%CI)**^▲^ | **Design Type** | **Usage** |
| --- | --- | --- | --- | --- | --- | --- | --- | --- | --- | --- | --- | --- |
| Inyang-Etoh PC, 2009^*^ | Adim village in Biase Local Government Area of Cross River State in southeastern Nigeria | Form August 2005 to June 2006 | schoolchildren, aged 4-20 yr, | *Sh* | Treated: a single oral dose of PZQ at 40 mg/kg, Control: placebo 40 mg/kg | Urine ﬁltration (10 ml × 2 specimens) | 8 weeks | 12/44 | 40/44 | 0.30 (0.18, 0.49) | RCT† | T |
| Touré S, 2008^*^ | Burkina Faso, western Africa | 2004-2006 | schoolchildren, aged 6-14 yr | *Sh* & *S. mansoni* | Treated: a single oral dose of PZQ at 60 mg/kg; Control: no drug given | Urine ﬁltration (10 ml; 1 specimen); Kato–Katz (2 slides × 1 specimen) | 1-2 years | 47/763 | 980/1644 | 0.10 (0.08, 0.14) | nRCT‡ | T |
| Tohon ZB, 2008^*^ | Niger | 2004 | Schoolchildren, aged 7, 8 and 11 yr | *Sh* & *S. mansoni* | Treated: a single oral dose of PZQ at 40 mg/kg; Control: no drug given | Urine ﬁltration (10 ml × 1 specimen); Kato–Katz (2 slides × 1 specimen) | 1 year | 546/1436 | 1230/1624 | 0.50 (0.47, 0.54) | nRCT | T |
| Saathoff E, 2004^*^ | KwaZulu/Natal, South Africa | From March 1998 to 1999 | Primary schoolchildren (age not speciﬁed) | *Sh* | Treated: a single oral dose of PZQ at 40 mg/kg; Control: no drug given | Urine ﬁltration (10 ml × 1 specimen) | 53 weeks | 237/825 | 754/1109 | 0.42 (0.38, 0.47) | nRCT | T |
| N'Goran EK, 2003^*^ | the village of Taabo, in south-central Côte d’Ivoire | Lasted for 8 months from November 2000 | primary schoolchildren, aged 5–15 yr | *Sh* | Treated: two doses of PZQ at 40 mg/kg by 4-week interval; Control: no drug given | Urine ﬁltration (10 ml × 2 specimens) | 8 months | 14/354 | 247/354 | 0.06 (0.03, 0.10) | nRCT | T |
| Borrmann S, 2001^*^ | Bindo, Tsouke´, and  Nombake´le´, in the province Moyen-Ogooue´, Gabon | From October 2000 to February 2001 | Children, aged 5-13 yr | *Sh* | Treated: a single oral dose of PZQ at 40 mg/kg; Control: one dose of PZQ placebo 40mg/kg | Urine ﬁltration (10 ml × 2 specimens) | 8 weeks | 24/89 | 24/30 | 0.34 (0.23, 0.50) | RCT | T |
| Campagne G, 2001^*^ | Niger | From 1995 to 1997 | Schoolchildren, aged 7–15 yr | *Sh* | Treated: a single oral dose of PZQ at 40 mg/kg | Urine ﬁltration (10 ml × 10 specimens) | over 34 months | 54/114 | 85/114 | 0.64 (0.51, 0.79) | nRCT | T |
| Utzinger J, 2000^*^ | Fagnampleu, western Côte d’Ivoire | From November 1998 to1999 | primary schoolchildren, aged 6 -14 yr | *S. mansoni* | Treated: two oral doses of PZQ at 30 mg/kg, administered 3h apart, and another dose at 40 mg/kg | Kato–Katz (2 slides × 4 specimens) | 63 days | 22/253 | 194/253 | 0.11 (0.08, 0.17) | nRCT | T |
| Nash TE, 1982^*^ | Bethesda, Maryland | Not reported | Patients, aged 4-47 yr | *Sj* &  *S. mekongi* | Treated: dosage of PZQ at 60 mg/kg in three divided doses | Kato–Katz (2 slides × 2 specimens) | 7 months | 1/11 | 11/11 | 0.13 (0.03, 0.59) | nRCT | T |
| Santos AT, 1979^*^ | Palo, Leyte, Philippines | Not reported | Patients, aged above 6 yr | *Sj* | Treated: a single oral dose of PZQ at 50 mg/kg, or 3×20 mg/kg given by 4-hour intervals; Control: placebo given similarly | Kato–Katz (2 slides × 3 specimens) | 12 months | 27/116 | 43/43 | 0.24 (0.17, 0.33) | RCT | T |
| McMahon JE, 1979^*^ | Tanga, Tanzania, Africa | Not reported | Schoolchildren, aged 7-15 yr | *Sh* | Treated: PZQ at 30 mg/kg, 40 mg/kg, 40 mg/kg in two divided doses by 4-hour apart; Control: placebo given similarly | Urine ﬁltration (10 ml × 3 specimens) | 6 months | (a) 6/28  (b) 8/28  (c) 6/31 | 30/30 | 0.23 (0.12, 0.45)  0.30 (0.17, 0.53)  0.21 (0.10, 0.41) | RCT | T |
| Katz N, 1979^*^ | military police of Minas Gerais, Brazil | Not reported | Male patients, aged 20-48 yr | *S. mansoni* | Treated: PZQ: (a) 1×20 mg/kg, (b) 2×20 mg/kg, and (c) 3×20 mg/kg; Control: placebo given similarly | Kato–Katz (2 slides × 3 specimens) | 6 -12 months | (a) 3/4  (b) 1/5  (c) 2/7 | 2/2  5/5  7/8 | 0.84 (0.39, 1.81)  0.27 (0.07, 1.11)  0.33 (0.10, 1.08) | RCT | T |
| Huang YX, 1998^*║^ | Jiangsu province, China | 1995 | People fighting against flood | *Sj* | Treated: a single oral dose of PZQ at 40 mg/kg at the 5^th^ week after contact; Control: no drug given | No | Half a year | 0/2250 | 45/60 | 0.00 (0.00, 0.00) | nRCT | P |
| Liu ZG, 1997^*║^ | Xiachong village, Yangxin County, Hubei province, China | August in 1995 | Villagers patients (age not speciﬁed) | *Sj* | Treated: (a) first dose of PZQ at 40 mg/kg 7 days after the first exposure and once every 14 days until 7 days after the last exposure; (b) PZQ at 40 mg/kg at 7^th^, 25^th^, 35^th^ day after first exposure; Control: no drug given | Kato–Katz (? slides × ? specimens) and miracidia hatching test | 28 days | (a) 0/85  (b) 3/70 | 59/61 | 0.01 (0.00, 0.10)  0.04 (0.01, 0.13) | RCT | P |
| Song Y, 2006^**^ | Jiangxi province, China | From May to November 2004 | Villagers, aged 6-65 yr | *Sj* | Treated:(a) AM at 6 mg/kg×13 doses by 15-day intervals; (b) AM at 6 mg/kg×7 doses by 1-month interval + placebo 6 mg/kg×6 doses by 15-day intervals; Control: placebo given similarly | Kato–Katz (4 slides × 1 specimen) | 40~60 days | (a) 14/413  (b) 42/402 | 79/587 | 0.25 (0.14, 0.44) 0.78 (0.55, 1.10) | RCT | P |
| Li YS, 2005^**^ | Poyang Lake region, Jiangxi province, China | From May to October in 2004 | Villagers, aged 6-60 yr | *Sj* | Treated: AM at 6 mg/kg×9~11 doses by 15-day intervals; Control: placebo given similarly | Kato–Katz (3 slides × 2 specimens) | 5 months | 3/373 | 56/361 | 0.05 (0.02, 0.16) | RCT | P |
| N’Goran EK, 2003^**^ | Taabo, the district of Tiassalé, Côte d’Ivoire | From November 2000 to July 2001 | schoolchildren, aged 6-15 yr | *Sh* | Treated: AM at 6 mg/kg×6 doses once every 4 weeks; Control: placebo given similarly | Urine ﬁltration (10 ml × 4 specimens) | 7 months | 76/156 | 97/150 | 0.75 (0.62, 0.92) | RCT | P |
| Utzinger J, 2000^**^ | Fagnampleu, western Côte d’Ivoire | From November 1998 to July 1999 | schoolchildren, aged 6-15 yr | *S. mansoni* | Treated: AM at 6 mg/kg×6 doses once every 3 weeks; Control: placebo given similarly | Kato–Katz (1 slides × 4 specimens) | 3 weeks | 31/128 | 68/140 | 0.50 (0.35, 0.71) | RCT | P |
| Tian ZY, 1999^**^ | Muping lake, China | From May to September in 1996 | Fishermen, aged 15-60 yr | *Sj* | Treated: AM at 6 mg/kg×8 doses once every 15 days; Control: placebo given similarly | Kato–Katz (3 slides × 1 specimen) | 1 month | 3/131 | 60/141 | 0.05 (0.02, 0.17) | RCT | P |
| Huang AS, 1999^**^ | The Yangtze River, China | From August to November in 1996 | People working with water (age not speciﬁed) | *Sj* | Treated: AM at 6 mg/kg×7 doses once every 2 weeks; Control: placebo given similarly | Kato–Katz (? slides × ? specimen) | 2 weeks | 2/41 | 3/24 | 0.39 (0.07, 2.17) | RCT | P |
| Song Y, 1998^**^ | Poyang Lake, Jiangxi Province, China | From mid-July to mid-August in 1996 | People fighting against floods (age not speciﬁed) | *Sj* | Treated: AM at 6 mg/kg once every 15 days×2~3 doses Control: placebo given similarly | Kato–Katz thick stool smears (? slides × ? specimen) | 40-50 days | 4/202 | 48/212 | 0.09 (0.03, 0.24) | RCT | P |
| Xu MS, 1997^**^ | Guichi, Anhui province, China | From April to November in 1996 | Villagers, aged 6-65 yr | *Sj* | Treated: AM at 6 mg/kg×10 doses once every 15days; Control: placebo given similarly | Kato–Katz (? slides × ? specimen) | 4 weeks | 0/433 | 40/452 | 0.01 (0.00, 0.21) | RCT | P |
| Tian ZY, 1997^**^ | Hanshou county, Hunan province, China | From June to October in 1996 | Villagers, aged 5-60 yr | *Sj* | Treated: AM at 6 mg/kg×9 doses once every 15 days; Control: placebo given similarly | Kato–Katz (3 slides × 1 specimen) and miracidia hatching test | 1 month | 5/290 | 82/305 | 0.06 (0.03, 0.16) | RCT | P |
| Wang JL, 1997^**^ | Yongsheng, Yunnan province, China | 1996 | Villagers, aged 3-60yr | *Sj* | Treated: AM at 6 mg/kg×10 doses once every half month; Control: placebo given similarly | Kato–Katz (3 slides × 1 specimen) and miracidia hatching test | 1 month | 23/789 | 87/717 | 0.24 (0.15, 0.38) | RCT | P |
| Xiao SH, 1996^**^ | Dali, Yunnan province, China | 1995 | Villagers, aged 4-60 yr | *Sj* | Treated: AM at 6 mg/kg×4 doses once every half month; Control: placebo given similarly | Kato–Katz (3 slides × 1 specimen) and miracidia hatching test | 25-32 days | 13/307 | 46/306 | 0.28 (0.16, 0.51) | RCT | P |
| Xiao SH, 1995^**^ | Yiyang, Hunan province, China | From August to October in 1994 | Villagers, aged 4-60 yr | *Sj* | Treated: AM at 6 mg/kg×4 doses once every half month; Control: placebo given similarly | Kato–Katz (3 slides × 1 specimen) and miracidia hatching test | 25-32 days | 20/365 | 51/376 | 0.40 (0.25, 0.66) | RCT | P |
| Inyang-Etoh PC, 2009^§^ | Adim village in Biase Local Government Area of Cross River State in southeastern Nigeria | From August 2005 to June 2006 | schoolchildren, aged 4-20 yr, | *Sh* | Treated: AS at 4 mg/kg daily for 3 consecutive days, Control: placebo given similarly | Urine ﬁltration (10 ml × 2 specimens) | 8 weeks | 13/44 | 40/44 | 0.33 (0.20, 0.52) | RCT | T |
| Borrmann S, 2001^§^ | Bindo, Tsouke´, and  Nombake´le´, in the province Moyen-Ogooue´, Gabon | From October 2000 to February 2001 | Children, aged 5-13 yr | *Sh* | Treated: PZQ placebo and AS at 4 mg/kg/day, once per day over 3 days, rounded to the nearest half; Control: AS placebo over 3 days and PZQ placebo once | Urine ﬁltration (10 ml × 2 specimens) | 8 weeks | 65/89 | 24/30 | 0.91 (0.73, 1.14) | RCT | T |
| Xu MS, 2001^§^ | Tangtian, Yanghe, Yinhui, and Shahe county, Anhui province, China | From April to October in 1996, and from July to September in 1997 to 1998 | People fighting against floods, fishermen, people living in boats, villagers, aged 6-65 yr | *Sj* | Treated: AS at 6 mg/kg once every 2 weeks since 7-10 days after exposure to 1 week later away from infected water; Control: placebo given similarly | Kato–Katz (? slides × ? specimen) and miracidia hatching test | 4 weeks | 9/2438 | 189/2660 | 0.05 (0.03, 0.10) | RCT | P |
| Cui JF, 2001^§^ | Taiyang village, Huihe County, Tongling City, Anhui province | From April 1998 to November 1999 | Villagers, aged 3-65 yr | *Sj* | Treated: AS at 6 mg/kg ×14 doses by 2-week interval since 7 days after exposure; Control: placebo given similarly | Kato–Katz (? slides × ? specimen) and miracidia hatching test | 4 weeks | 0/305 | 16/305 | 0.03 (0.00, 0.50) | RCT | P |
| Sun MX, 2000^§^ | Tongling, Anhui province, China | From April 1998 to November 1999 | High risk villagers | *Sj* | Treated: AS at 6 mg/kg×14 doses by 2-week intervals; Control: placebo given similarly | Kato–Katz (? slides × ? specimen) | 4 weeks | 1/208 | 18/207 | 0.06 (0.01, 0.41) | RCT | P |
| Zhang SJ, 2000^§^ | Poyang lake, Jiangxi province; Guichi and Wangjiang county, Anhui province; Jiayu county, Hubei province | From 1993 to 1998 | Villagers, aged 6-65yr | *Sj* | Treated: (a) AS at 6 mg/kg×8 doses by 7-day intervals; AS at 6 mg/kg by 15-day intervals: (b): 5 doses, (c): 3 doses; Control: placebo given similarly | Kato–Katz (? slides × ? specimen) and miracidia hatching test | 30 days | (a1) 0/185  (a2) 0/56  (b1) 1/132  (b1) 4/151  (c) 6/51 | 7/166  8/52  7/148  13/156  12/64 | 0.06 (0.00, 1.04)  0.05 (0.00, 0.92)  0.16 (0.02, 1.28)  0.32 (0.11, 0.95)  0.63 (0.25, 1.56) | RCT | P |
| Yi ZH, 2000^§^ | Jiangling county, Hubei province, China | From July to August of both in 1997 and 1998 | Both farmers, aged 18-38 yr and soldiers fighting against flood aged 18-32 yr | *Sj* | Treated: AS at 6 mg/kg×3 doses at the 7^th^, 14^th^ and 15^th^ day after exposure; Control: placebo given similarly | Miracidia hatching test | 4 weeks | 2/168 | 22/200 | 0.11 (0.03, 0.45) | RCT | P |
| Liu HY, 1999^§^ | Xinjian county, Jiangxi province, China | From June to November in 1997 | Villagers, aged 6-65 yr | *Sj* | Treated: AS at 6 mg/kg×13 doses by 15-day intervals; Control: placebo given similarly | Kato–Katz (? slides × ? specimen) and miracidia hatching test | 30 days | 0/43 | 4/58 | 0.15 (0.01, 2.70) | RCT | P |
| Xu MS, 1998^§^ | Wangjiang county, Anhui province, China | From July to September in 1997 | Villagers, aged 6-65 yr | *Sj* | Treated: AS at 6 mg/kg×4 doses by 2-week intervals; Control: placebo given similarly | Kato–Katz (? slides × ? specimen) and miracidia hatching test | 4 weeks | 2/380 | 18/400 | 0.12 (0.03, 0.50) | RCT | P |
| Liu ZD, 1996^§^ | Poyang Lake, Jiangxi province, China | Summer in 1994 | Villagers (age not speciﬁed) | *Sj* | Treated: AS at 6 mg/kg×8 doses by 1-week interval; Control: placebo given similarly | Kato–Katz (? slides × ? specimen) and miracidia hatching method | 4 weeks | 0/226 | 26/179 | 0.01 (0.00, 0.24) | RCT | P |
| Xu MS, 1996§ | Tangtian county, Guichi, Anhui province, China | From April to November in 1996 | Villagers, aged 6-65 yr | *Sj* | Treated: AS at 6 mg/kg×10 doses by half a month interval; Control: placebo given similarly | Kato–Katz (? slides × ? specimen) and miracidia hatching test | 4 weeks | 1/323 | 31/323 | 0.03 (0.00, 0.23) | RCT | P |
| Wu LJ, 1995^§^ | Xinjian county and Jinxian county, Jiangxi province, China | Autumn in 1993 | Villagers, aged 10-70 yr | *Sj* | Treated: AS at 6 mg/kg×8 doses by 1-week interval; Control: placebo given similarly | Kato–Katz (3 slides × 1 specimen) and miracidia hatching test | 4 weeks | 0/346 | 15/323 | 0.03 (0.00, 0.50) | RCT | P |
| Hou XY,  2008^††^ | Dongting Lake region, Hunan province, China | From May 2003 to December 2005 | Patients (farmer, fisherman, businessman, student), aged 10–60 yr | *Sj* | Treated: 60 mg/kg PZQ+6 mg/kg AM once; Control: 60 mg/kg PZQ+AM placebo once | Kato–Katz (? slides × ? specimen) and miracidia hatching test | 45 days | 2/95 | 4/101 | 0.54 (0.05, 5.77) | RCT | T |
| Inyang-Etoh PC, 2009^‡‡^ | Adim village in Biase Local Government Area of Cross River State in southeastern Nigeria | From August 2005 to June 2006 | schoolchildren, aged 4-20 yr | *Sh* | Treated: PZQ at 40 mg/kg once and AS at 4 mg/kg daily for 3 consecutive days; Control: AS placebo at 4 mg/kg for 3 consecutive days and PZQ at 40mg/kg once | Urine ﬁltration (10 ml × 2 specimens) | 8 weeks | 5/44 | 12/44 | 0.42 (0.16, 1.08) | RCT | T |
| Borrmann S, 2001^‡‡^ | Bindo, Tsouke´, and  Nombake´le´, in the province Moyen-Ogooue´, Gabon | From October 2000 to February 2001 | children aged 5-13 yr | *Sh* | Treated: PZQ at 40 mg/kg once and AS at 4 mg/kg/day×3; Control: PZQ at 40 mg/kg once, rounded to the nearest half and AS placebo at 4 mg/kg/day×3 | Urine ﬁltration (10 ml × 2 specimens) | 8 weeks | 17/88 | 24/89 | 0.72 (0.41, 1.24) | RCT | T |
| Xia CS, 2000^‡‡^ | Yangtze River region, China | From July to September in 1998 | Soldiers fighting against floods (age not speciﬁed) | *Sj* | Treated: 300mg AS by 1-week interval and 1200mg PZQ divided into two doses; Control: no drugs received | Immunological test for schistosomal antigen and antibody | 30-40 days | 2/1362 | 4/112 | 0.04 (0.01, 0.22) | nRCT | P |

* Studies of PZQ efficacy for schistosomiasis control

† RCT denotes randomized controlled trial.

‡ nRCT denotes non-randomized controlled trial.

§Studies of AS efficacy for schistosomiasis control.

║ The two studies were separately included in this meta-analysis because of their different purpose from other studies of PZQ.

** Studies of AM efficacy for preventing schistosomiasis

†† Study of PZQ and AM in combination for treating schistosomiasis

‡‡ Studies of PZQ with AS in combination for schistosomiasis control

△ n/N=number diagnosed as positive over number of participants diagnosed

▲95% CI=95 percent confidence interval. *Sh*=*Schistosoma haematobium*, *Sj*=*Schistosoma japonicum*, P=Prevention, T=Treatment, yr=years old
